# Plant growth-promoting rhizobacteria enhanced induced systemic resistance of tomato against *Botrytis cinerea* phytopathogen

**DOI:** 10.3389/fpls.2025.1570986

**Published:** 2025-04-15

**Authors:** Ismael Mazuecos-Aguilera, Francisco Anta-Fernández, Andrea Crespo-Barreiro, Alejandro Martínez-Quesada, Luis Lombana-Larrea, Fernando González-Andrés

**Affiliations:** ^1^ Chemical, Environmental and Bioprocess Engineering Group, Institute for Research and Innovation in Engineering (I4), University of León, León, Spain; ^2^ FICOSTERRA, Burgos, Spain

**Keywords:** PGPR, biocontrol, *Botrytis cinerea*, tomato, induced systemic resistance, sustainable agriculture

## Abstract

**Introduction:**

*Botrytis cinerea* is one of the pathogenic fungi causing major problems worldwide in crops such as tomato. Some Plant Growth-Promoting Rhizobacteria (PGPR) can activate induced systemic resistance (ISR) pathways in crops, reducing the need for antifungals.

**Methods:**

Three strains belonging to the species *Peribacillus frigoritolerans* (CD_FICOS_02), *Pseudomonas canadensis* (CD_FICOS_03), and *Azotobacter chroococcum* (CD_FICOS_04), which exhibit outstanding PGPR properties, were evaluated for their ability to protect tomato plants against *B. cinerea* infection by ISR via soil inoculation.

**Results:**

The strains CD_FICOS_02 and CD_FICOS_03 reduced *B. cinerea* incidence and plant oxidative stress. The first strain mainly increased the expression of genes related to the salicylic acid pathway, while the second increased the expression of genes related to the jasmonic acid/ethylene hormonal pathway, indicating preferential ISR activation by each of these pathways. In addition, CD_FICOS_03 was able to increase the root and aerial biomass production of infected plants compared to the control. Interestingly, although the strain CD_FICOS_04 did not reduce the damage caused by *B. cinerea*, it increased the biomass of infected plants.

**Discussion:**

Our results suggest that the best strategy for biocontrol of *B. cinerea* is to combine the ability to promote plant growth with the ability to induce systemic resistance, as demonstrated by strains *P. frigoritolerans* CD_FICOS_02 and *P. canadensis* CD_FICOS_03.

## 1 Introduction

The tomato (*Solanum lycopersicum* L.) stands as one of the most globally significant vegetables, trailing only potatoes in both production and consumption (Li et al., 2023; Roșca et al., 2023). FAOSTAT data for 2022 reported a harvest of approximately 186,107,972 tons of tomatoes across 4,917,735 hectares worldwide (FAOSTAT, 2024). However, tomato cultivation faces the persistent challenge of gray mold, a necrotic disease caused by *Botrytis cinerea* (Tian et al., 2023; Zheng et al., 2023). *B. cinerea* is the second most important pathogenic fungus from a scientific and economic point of view, capable of infecting over 200 plant species and causing global losses exceeding 10 billion dollars (Dean et al., 2012; Tian et al., 2023). In the perpetual battle between plants and *B. cinerea*, the fungus inflicts damage upon plant cells by secreting toxic compounds such as botcinolide, botrydial or oxalic acid and by inducing the production of reactive oxygen species (ROS), which can impede host defenses (Van Kan, 2005; Tian et al., 2023). Conversely, plants deploy a primary line of structural defense composed of cuticular wax and the cell wall, shielding the leaf and individual cells, respectively. Beyond these structural defenses, plants activate more sophisticated immune responses, such as systemic acquired resistance (SAR) and induced systemic resistance (ISR) (Qi et al., 2018). Via SAR, the plant recognizes microbial-associated molecular patterns (MAMPs), activating MAMP-triggered immunity (MTI); or pathogen effectors (avirulence protein, Avr), activating effector-triggered immunity (ETI). MTI and ETI extend beyond local infection sites, manifesting as broad-spectrum resistance (Qi et al., 2018). MTI can also be induced by interactions with non-pathogenic agents that colonize host plant tissues, provoke the activation of ISR, triggering the expression of defense genes from various hormonal pathways. In this way SAR would be triggered by pathogens, among other stress factors, and ISR by beneficial microorganisms (Qi et al., 2018). Some of these non-pathogenic agents can be Plant Growth-Promoting Rhizobacteria (PGPR), which in addition to activating ISR can stimulate plant growth by producing bioactive substances and improving nutrient availability (Poveda et al., 2020).

Classically, SAR signaling has been associated with salicylic acid (SA)-dependent and ISR dependent on jasmonic acid (JA) and ethylene (ET). Nevertheless, in many cases an induction of ISR via the SA hormonal pathway has also been observed. Thus SA, JA and ET are known to play a pivotal role in the plant defence response, however, many aspects of their interaction and regulation of diverse defense proteins and genes are still not well understood (AbuQamar et al., 2017; Poveda et al., 2020; Tsalgatidou et al., 2023). Another type of response would be the priming phenomenon, which refers to the enhancement of a plant’s ability to respond more quickly and efficiently to future pathogen attacks or environmental stress, which may involve priming both SAR and ISR. This process is essential for improving plant immunity, making it more efficient and less energy-consuming, as it prepares the plant to activate defence mechanisms more quickly upon pathogen recognition (Tsalgatidou et al., 2023).

*Bacillus* and *Pseudomonas* are the bacterial genera most frequently associated with *B. cinerea* biocontrol. However, numerous other genera can function as both PGPR and biocontrol agents simultaneously (Poveda et al., 2020; Tsalgatidou et al., 2023). Implementing ISR-activating PGPR can enhance plant growth by optimizing nutrient availability and utilization while effectively controlling pathogens like *B. cinerea*. This approach also has the potential to reduce reliance on costly chemical fertilizers and fungicides, providing both economic and environmental benefits (Dean et al., 2012; Zheng et al., 2021; Mazuecos-Aguilera et al., 2024). The aim of this work was to evaluate the ability of three PGPR strains belonging to the species *Pseudomonas canadensis*, *Peribacillus frigoritolerans* and *Azotobacter chroococcum*, to induce ISR, analyzing the hormonal activation pathways involved and their effectiveness in the control of *B. cinerea* in tomato plants, in terms of reduction of disease incidence and improvement of overall plant health. For this purpose, tomato plants were inoculated at soil level with the strains selected on the basis of characteristics other than biocontrol and the leaves were infected with *B. cinerea*. The evaluation consisted of assessing the progression of infection in the different treatments, the hydrogen peroxide and malondialdehyde content, the biomass produced by the plants and the expression of genes involved in ISR was assessed in order to elucidate the molecular mechanisms underlying the plant-bacteria-pathogen interaction.

## 2 Materials and methods

### 2.1 Microbiological material

The strains selected for this work were as follows: *Peribacillus frigoritolerans* CD_FICOS_02 isolated from soil samples of pepper crops in Zamora (Spain), *Pseudomonas canadensis* CD_FICOS_03 and *Azotobacter chroococcum* CD_FICOS_04 both isolated from citrus crop soils located in the Valencian Community (Spain). The strains were selected for their different PGPR properties *in-vitro* and *in-planta* evaluated in previous assays among a broader battery of promising PGP bacteria. All these strains are cited in this work for the first time and are registered in the Spanish Type Culture Collection (Colección Española de Cultivos Tipo, CECT) by Ficosterra S.L. for patent purposes (CECT no. 30923, CD_FICOS_02; 30930, CD_FICOS_03; 30924, CD_FICOS_04).

Taxonomic identification of the strains involved amplification and sequencing of the 16S rRNA gene by Macrogen (The Netherlands), following the methodology outlined by Marcano et al. (2016). The obtained sequences were processed and deposited at GenBank (accession no. PQ061804, *P. frigoritolerans* CD_FICOS_02; PQ061494, *P. canadensis* CD_FICOS_03; PQ061500 *A. chroococcum* CD_FICOS_04) and compared with those from the EzTaxon-e server, which contains the type strains of all described bacterial species (Kim et al., 2012).

The bacterial strains *P. canadensis* CD_FICOS_03 and *P. frigoritolerans* CD_FICOS_02 were isolated and maintained on tryptic soy agar (TSA, Sigma-Aldrich, Madrid, Spain), while Ashby-mannitol agar was used for *A. chroococcum* CD_FICOS_04. The Ashby-mannitol agar media (1 L water) contained mannitol (5 g), K2HPO4 (0.2 g), MgSO_4_.7H_2_O (0.2 g), CaSO4 (0.1 g), NaCl (0.2 g), CaCO_3_ (0.1 g) and agar (15 g).

*Botrytis cinerea* (CECT 20973), obtained from the Spanish Type Culture Collection (CECT; Valencia, Spain), was cultured on potato-dextrose agar (PDA, Sigma-Aldrich, Madrid, Spain) supplemented with powdery tomato leaves (0.1% v/v) to maintain its phytopathogenic activity (Poveda et al., 2022). *B. cinerea* strain identification was carried out both morphologically and molecularly by sequencing of the ITS region. The sequence obtained was deposited in GenBank (accession no. PV225659).

### 2.2 Determination of plant growth-promoting traits *in-vitro*

For each of the strains included in the study, different assays were carried out to evaluate their Plant Growth-Promoting traits. All tests were carried out in triplicate. In addition, for the tests carried out on plates, each replicate (plate) contained three replicates (colonies).

#### 2.2.1 *In-vitro* N fixation capacity

The N fixation capacity of the strains was assessed via the acetylene reduction assay (ARA) following the method described by Habibi et al. (2014). For this purpose, 20 mL chromatography vials filled with 10 mL culture medium without nitrogen are inoculated with the strain to be evaluated until a low (OD600nm of 0.1) and high (OD600nm of 0.6) cell density is reached. Subsequently, 10% of the gas space in each vial is replaced with pure acetylene gas and the vials are incubated at 30°C for 20 days with orbital shaking at 170 rpm. During this time, ethylene production is measured at 3, 7, 13 and 20 days after inoculation, using 2% (v:v) ethylene as a standard. The concentration of ethylene in the vial was determined as described in Mazuecos-Aguilera et al. (2024) using an HP-5890 gas chromatograph (Hewlett Packard, Wilmington, DE) equipped with a Poropak R 80/100 mesh column and an FID detector.

#### 2.2.2 Siderophore production

Siderophore production was assessed using the methodology of Alexander and Zuberer (1991) with the chromium azurol S agar (CAS) medium described by Schwyn and Neilands (1987). CAS agar plates were inoculated with the different strains and incubated at 28°C for 72 hours. The presence of orange halos around colonies on blue agar indicated excretion of siderophores. The siderophore production rate was calculated by dividing the halo diameter by the colony diameter.

#### 2.2.3 Indole-3-acetic acid production

For each isolate, a suspension of 3×10^8^ CFU/mL in sterile saline was prepared from the bacterial growth in a Petri dish with TSA medium. 500 µL of this suspension was then transferred to a test tube containing 4 mL of TSB (SIGMA, catalogue no. T8907) and incubated at 28°C with continuous shaking for 3 days. Then, 500 µL of the incubated suspension was transferred to a test tube containing 4 mL of JMM medium (O’Hara et al., 1989) supplemented with 0.167 g/L L-tryptophan and incubated for another 4 days at 28°C with continuous shaking. After incubation, the bacterial concentration was estimated by measuring the optical density at 600 nm. Then, 1.5 mL of the suspension was centrifuged at 2500 g for 10 minutes. One millilitre of Salkowski’s reagent (0.01 M FeCl₃ and 34.3% perchloric acid) was added to 1 mL of the supernatant and incubated in the dark for 30 minutes. IAA production was determined by measuring the optical density at 535 nm and comparing it to a standard curve of known IAA concentrations.

#### 2.2.4 1-aminocyclopropane-1-carboxylate deaminase activity

The ACC-deaminase activity of the isolated strains was assessed using Dworkin-Foster (DF) minimal medium (Dworkin and Foster, 1958) supplemented with 1-aminocyclopropane-1-carboxylate (ACC) as the sole nitrogen source (Penrose and Glick, 2003). The plates were incubated in the dark at 28°C for three days. Bacterial growth on DF agar medium containing ACC was considered indicative of potential ACC-deaminase activity, as only bacteria capable of utilizing ACC as a nitrogen source could proliferate. For quantitative analysis of ACC-deaminase activity, the protocol described by El-Tarabily (2008) was followed. After measuring protein content and α-Ketobutyrate (α-KB) levels, enzyme activity was expressed as micromoles of α-KB per milligram of protein per hour in the active isolates.

#### 2.2.5 Phosphate solubilization

Phosphate solubilization capacity was evaluated qualitatively and quantitatively. For qualitative evaluation, the methodology described in Marcano et al. (2016) was followed. Each pure isolate was grown in a Petri dish containing YED-P medium and incubated at 30°C for 10 days. The formation of a clear halo around the bacterial colonies indicated phosphate solubilization. P solubilization was measured by the ratio of halo to colony diameter. The YED-P media (1L) contains yeast extract (2 g), glucose (10 g), Ca_3_(PO_4_)_2_ (4 g) and agar (12 g). For quantitative assessment of phosphate solubilization 40 ml of liquid NBRIP medium was inoculated with 160 μl of inoculum of each strain and incubated at 30°C, 120 rpm/min for 7 days. 1.5 ml of each culture was taken every 24 hours. Centrifuged at 10,000 rpm for 15 minutes. 1 ml of supernatant was taken to which 9 ml distilled water was added to estimate the soluble phosphate content by the Molybdenum Blue colorimetric method described by Murphy and Riley (1962). The NBRIP media (1L) contains glucose (10 g), MgCl_2_·6H_2_O (5 g), MgSO_4_·7H_2_O (0.25 g), KCl (0.2 g), (NH_4_)_2_SO_4_ (0.1 _g_) and Ca_3_(PO_4_)_2_ (5 g).

#### 2.2.6 Potassium solubilization

The solubilization capacity of inorganic potassium was determined using Aleksandrov media plates modified as described by Rajawat et al. (2016), prepared by using mica (potassium aluminum silicate) as the source of insoluble potassium. The halo zone with color change was noted for K solubilization. The Aleksandrov-modified media (1 L water) contained glucose (5 g), MgSO_4_.7H_2_O (0.5 g), FeCl_3_ (0.005 g), Ca_3_(PO_4_)_2_ (2 _g_), AlKO_6_Si_2_ (2 g), Agar (15 g), CaCO_3_ (0.1 g) and bromothymol blue (20 ml from a Stock 50 mg/10ml ethanol 70%).

### 2.3 Determination of plant growth-promoting traits *in-planta*

Prior to the in-plant assay on tomato, a plant assay on barley under hydroponic conditions was carried out to evaluate the in-plant PGP capacity of the strains used.

Barley seeds var. Scarlett were disinfected by washing with 70% ethanol (1 min) and 1.5% sodium hypochlorite (5 min) followed by rinsing three times with sterile distilled water. The seeds were then pregerminated in darkness. Germinated seeds were sown in 1 L pots with washed and sterile vermiculite. Each seed was inoculated with 1 mL of a bacterial suspension at 10^8^ CFU ml^-1^ at the time of sowing. The bacterial suspension was obtained by culture in tryptic soy broth (TSB) for *P. canadensis* and *P. frigoritolerans* or Ashby-mannitol medium for *A. chroococcum* (120 rpm, 24-72 h, 28°C). Bacterial concentration was measured by decimal dilution and plate seeding and the cultures were diluted to the concentration of 10^8^ CFU ml^-1^. Two experiments were performed in a phytotron with controlled conditions in a completely randomized design. The number of biological replicates per treatment and experiment was 5. The phytotron conditions were as follows: photoperiod of 14 h day (22 ± 2°C), 10 h nigh (16 ± 2°C) with a homogenised light intensity for each plant, and relative humidity of 60 ± 6%. Plants were watered weekly with 150 mL of distilled water and every 15 days with sterile Rigaud and Puppo solution. The Rigaud and Puppo solution (1 L water) contained EDTA FeNa_2_ (25 mg), Na_2_MoO_4_ (4.7 mg), H_3_BO_4_ (18 mg), MnSO_4_ (20 mg), ZnSO_4_ (3 mg), CuSO_4_ (2 mg), KH_2_PO_4_ (200 mg), KCl (200 mg), MgSO_4_ (200 mg) and NH_4_NO_3_ (400 mg). After 4 weeks, chlorophyll content was determined using the portable SPAD-502 apparatus (Minolta Corp., Japan) after which the plants were harvested and the dry weight of aerial and root parts was measured. A two-way analysis of variance (ANOVA) was performed with SPSS Statistics v.29.0.2.0 software appropriate for a complete randomized design with experiment and treatment as the independent variables. Dunnet’s *post-hoc* test against the uninoculated control was used to identify significant differences between means.

### 2.4 *In-planta* biocontrol of *Botrytis cinerea* assay

#### 2.4.1 Plant material and growing conditions

Seeds of tomato var. Raf were disinfected by washing with 70% ethanol (1 min) and 1.5% sodium hypochlorite (5 min) followed by rinsing three times with sterile distilled water.

Seeds were germinated in seedbeds and transplanted into 1 L pots, containing a sterilized professional substrate/vermiculite mixture (3:1). The plants were maintained in a greenhouse.

#### 2.4.2 Bacterial inoculation and fungal infection

For each strain, a bacterial inoculum was prepared by growth in tryptic soy broth (TSB), for *P. canadensis* and *P. frigoritolerans*, or Ashby-mannitol medium, for *A. chroococcum* (120 rpm, 24-72 h, 28°C). Subsequently, bacterial concentration was determined by decimal dilution and plating and the cultures were diluted to a concentration of 10^7^ CFU ml^-1^. Tomato seedlings var. Raff were treated by applying a 1 ml suspension of each bacterium to individual roots when the plants had two true leaves.

The fungal infection was conducted by applying a disk of PDA media containing actively growing *B. cinerea* (from the periphery of the plate) to the second leaflet of the fourth and fifth oldest leaf of the plant, avoiding the main nerves and exposing the mycelium of the fungus directly to the leaf. Infection was carried out when the plants had at least 6 true leaves. After that the plants were covered with a plastic bag to maintain a constant high humidity and favor infection by the fungus.

#### 2.4.3 Experimental design

Two independent experiments were conducted, each with a completely randomized design. The number of biological replicates was 5 per experiment and treatment. The treatments are described in **Table 1**. The experiments were performed in a phytotron with controlled conditions as follows: photoperiod of 14 h day at 22°C ± 2°C and 10 h night at 16°C ± 2°C with a homogenized light intensity of 10,000 lm/m^2^ for each plant; relative humidity was 60 ± 6%. After transplanting the tomato seedling, the treatments were applied as follows: at the stage of two true leaves, the seedlings were inoculated with the corresponding PGPR, namely *P. frigoritolerans* CD_FICOS_02, *P. canadensis* CD_FICOS_03, *A. chroococcum* CD_FICOS_04, plus a non-inoculated control group. Subsequently, when the plants reached the stage of six true leaves, the treatments infected with *B. cinerea* received the disk of PDA media containing the pathogen as described in section 2.4.2. Three days after infection, infected leaflets, or their counterparts on non-infected plants, were collected, immediately photographed, rapidly stored in liquid nitrogen and preserved at -80 °C for further analysis of H_2_O_2_ and MDA content and gene expression.

**Table 1:** Description of the eight treatments evaluated in the *in-planta* assay.

Nomenclature treatment	Treatment	Number of biological replicates per treatment and experiment
Control	Plants not infected with *B. cinerea* or inoculated with bacteria	5
CD_FICOS_02	Plants not infected with *B. cinerea*, but inoculated with CD_FICOS_02	5
CD_FICOS_03	Plants not infected with *B. cinerea*, but inoculated with CD_FICOS_03	5
CD_FICOS_04	Plants not infected with *B. cinerea*, but inoculated with CD_FICOS_04	5
Control *Botrytis*	Plants infected with *B. cinerea*, but not inoculated with bacteria	5
CD_FICOS_02 *Botrytis*	Plants infected with *B. cinerea* and inoculated with CD_FICOS_02	5
CD_FICOS_03 *Botrytis*	Plants infected with *B. cinerea* and inoculated with CD_FICOS_03	5
CD_FICOS_04 *Botrytis*	Plants infected with *B. cinerea* and inoculated with CD_FICOS_04	5

Two independent experiments were conducted.

#### 2.4.4 Aerial and root biomass

The aerial part of the plants was harvested at the base of the stem and the fresh biomass was quantified. The roots were carefully washed to eliminate any residual substrate, and after excess water was removed by drying, the fresh biomass was determined. Both the aerial part and root were then subjected to drying at 70°C for 96 hours followed by measurement of the dry biomass.

#### 2.4.5 *Botrytis cinerea* lesion evaluation

Photographs of the second leaflets of the fourth and fifth oldest leaf of 5 infected plants per treatment and per experiment and 5 non-infected plants per treatment and per experiment were processed and analyzed to assess the extent of infection damage. The total area and the injured area of the photographed leaflets was calculated employing Fiji software v.2.2.0 (ImageJ) (Schindelin, 2012). Then the relative percentage of injured area relative to the total leaflet area was calculated according to the following formula:


$$Relative\;percentage\;of\;injured\;area=\frac{\left(Injured\;area\cdot 100\right)}{Total\;area\;of\;leaflet}$$

To evaluate the distribution of data across treatments, a combined boxplot and kernel density estimation (KDE) plot was created. Both methods were combined into a single plot to facilitate visual comparison between treatments. The plot was generated using the ggplot2 package in R (Wickham and Sievert, 2009).

#### 2.4.6 Defense gene expression in plants

To assess the impact of PGPR inoculation and *B. cinerea* infection on the induction of systemic resistance phenomena, we conducted an analysis of defense gene expression using quantitative reverse transcription polymerase chain reaction (RT-qPCR). RNA extraction was carried out from infected leaflets and their counterparts in uninfected plants following the protocol of the commercial Direct-zolTM RNA Miniprep Kit (Zymo Research, Irvine, CA, USA), with TRIzol reagent (Invitrogen, Waltham, Massachusetts, USA) and including the DNase column treatment step. RNA was extracted from three biological replicates per treatment in each experiment, each biological replicate consisting of one leaflet from three to four tomato plants. Samples with remaining DNA were treated with DNase using the TURBO DNA-free kit (Thermo Fischer Scientific, MA, USA). Subsequently, RNA concentration and integrity were checked by agarose gel electrophoresis and by Nanodrop quantification using a NanoDrop 2000/2000c spectrophotometer (NanoDrop Technologies; Thermo Fisher Scientific, Inc., Pittsburgh, PA, USA). cDNA was synthesized from RNA with Reverse Transcription System (Promega, Madison, WI, USA).

Gene expression was analysed by RT-qPCR using an Agilent Mx3005P Real-Time PCR System (Agilent Technologies, Santa Clara, CA, USA) with Luna ^®^ Universal qPCR Mastermix (New England Biolabs, Ipswich, MA, USA). All PCR reactions were performed in triplicate in a total volume of 10 μL following the manufacturer’s recommendations. Threshold cycles (CT) were calculated using the tomato Actin gene as an endogenous control. The primers used were described in Poveda et al. (2019) and are shown in **Supplementary Table S1**. Data are expressed using the 2−ΔΔCT method (Livak and Schmittgen, 2001).

#### 2.4.7 Hydrogen peroxide content

The levels of hydrogen peroxide (H_2_O_2_) were quantified in both infected leaflets and their counterparts in non-infected plants following the methodology described in Crespo-Barreiro et al. (2025). The experiment was conducted with three biological replicates per treatment in each experiment, and each biological replicate was a pool of one leaflet obtained from 3-4 plants. Leaf tissues (100 mg) were homogenized in an ice bath with 1 ml of 0.1% (w/v) trichloroacetic acid (TCA). After homogenization, the mixture was centrifuged at 12,000 × g for 15 minutes. Then, 0.5 ml of the obtained supernatant was mixed with 0.5 ml of 10 mM potassium phosphate buffer (pH 7.0) and 1 ml of 1 M potassium iodide (KI). The reaction was carried out in darkness for 1 hour, following the method described by Alexieva et al. (2001). Finally, the absorbance of the supernatant was measured at 390 nm to determine H_2_O_2_ content, using a standard curve method as outlined by Velikova et al. (2000).

#### 2.4.8 Malondialdehyde content

The evaluation of lipid peroxidation in both infected leaflets and their counterparts in non-infected plants was performed using the thiobarbituric acid (TBA) test, following the protocol of Heath and Packer (1968) and detailed by Velikova et al. (2000), focusing on malondialdehyde (MDA) content as an indicator of lipid peroxidation. Leaf samples (100 mg) were homogenized in 1 ml of a 0.1% (w/v) trichloroacetic acid (TCA) solution and centrifuged at 10,000 × g for 20 minutes. Subsequently, 0.5 ml of the supernatant was mixed with 1 ml of 0.5% (w/v) TBA in 20% TCA. The mixture was incubated in a boiling water bath for 30 minutes and then cooled in an ice bath to stop the reaction. After centrifugation at 10,000 × g for 5 minutes, the absorbance of the supernatant was measured at 532 nm, with non-specific absorption at 600 nm subtracted. The amount of MDA–TBA complex (red pigment) was determined using the extinction coefficient of 155 mM⁻¹ cm⁻¹, calculating the MDA content (nmol/ml FW) using the formula:


$$MDA\;(\frac{nmol}{ml}FW)\;=\;[(A\;532nm\;-\;A\;600nm\;)/155000]\;\times \;1,000,000.$$

#### 2.4.9 Statistical analysis

Dependent variables that met the assumptions of normality and homogeneity of variances, assessed by the Shapiro-Wilk test and the Levene test, respectively, were analyzed by analysis of variance (ANOVA), while nonconforming variables, namely the variable lesion, was analyzed by the nonparametric Kruskal-Wallis test. The ANOVA performed was the appropriate for a completely randomised design with experiment and treatment as independent variables. For the dependent variables fresh and dry biomass of aerial part and roots, a separate ANOVA was performed for the uninfected and infected treatment groups. A Dunnett’s test was then performed for the variable treatment, using the Control or Control Botrytis treatments as the respective control group. The analysis was designed to evaluate the effect of inoculation rather than the effect of *B. cinerea* on biomass production. For the dependent variable gene expression, H_2_O_2_ levels, and MDA levels, a pooled ANOVA was performed for the uninfected and infected treatment groups. This was followed by a Tukey test for the variable treatment. The analysis was designed to evaluate the effect of both inoculation and infection on these variables. For lesion variable non-parametric Kruskal-Wallis tests was used because the data did not meet the assumptions of normality and/or homogeneity of variances, with one test for the treatment factor and another for the experimental factor. SPSS Statistics v.29.0.2.0 software was used for all analyses.

## 3 Results

### 3.1 Plant growth-promoting capabilities of selected isolates

Evaluation of the Plant Growth-Promoting (PGP) abilities of the strains revealed favorable characteristics across all three isolates. Strain CD_FICOS_04 exhibited significant capacity to reduce ethylene, indicating its potential as an efficient atmospheric nitrogen fixer, as determined by the acetylene reduction assay (ARA) method (**Table 2**). All three strains showed similar values for insoluble P solubilization and siderophore production (**Table 2**). Notably, strains CD_FICOS_02 and CD_FICOS_03 exhibited remarkable 1-aminocyclopropane-1-carboxylate (ACC) deaminase enzyme activity (**Table 2**). Moreover, all strains showed IAA production capacity, with the highest value observed in strain CD_FICOS_02 (**Table 2**). Furthermore, all three strains exhibited potassium solubilization capacity, with strain CD_FICOS_03 demonstrating the highest intensity (**Table 2**).

**Table 2:** Details of isolate, closest relative and physiological characteristics.

Isolate name	Closest relative based on 16S rRNA gene sequence	ARA^a^	P solubilisation		Siderophore production	IAA production	ACC deaminase activity ^c^	K solubilisation
Units	% similarity	nmol ethylene h^−1^	index^b^	µg P/ml	index^b^	µg ml^-1^	µmol α-ketobutyrate mg^-1^ h^-1^	intensity of colour change^d^
CD_FICOS_02	*Peribacillus frigoritolerans* (97.17)		2.22	56.61	2.34	9.72	115.32	++
CD_FICOS_03	*Pseudomonas canadensis* (100)		3.13	63.54	2.68	5.61	99.29	++++
CD_FICOS_04	*Azotobacter chroococcum* (99.15)	10937	2.41	64.41	2.86	4.64	15.90	+++

a Acetylene reduction assay (ARA).

b Index = mm halo diameter/mm colony diameter.

c 1-aminocyclopropane-1-carboxylate (ACC).

d Qualitative evaluation of potassium solubilization capacity is based on the size and intensity of the yellow shift on Aleksandrov’s agar medium.

In the barley plant test conducted under hydroponic conditions, prior to the tomato assay, ANOVA showed that the experimental variable did not produce significant differences in the dependent variables. Whereas the treatment with the inoculation of PGPR strains produced significant differences on aerial and root biomass and chlorophyll content (**Supplementary Table S2**). No significant interactions between experiment and treatment were detected (**Supplementary Table S2**). CD_FICOS_02 enhanced root dry weight compared to the non-inoculated control. CD_FICOS_03 increased both aerial and root dry weight, as well as chlorophyll content compared to the control. And CD_FICOS_04 promoted an increase in both aerial and root dry weight (**Supplementary Figure S1; Supplementary Table S3**).

### 3.2 Biomass in tomato plants

ANOVA showed that the variable experiment did not produce significant differences in any of the dependent variables, while treatment with the PGPR strain resulted in significantly higher biomass production in some of the treatments infected with *B. cinerea*, but not in those not infected with the pathogen (**Supplementary Tables S4**, **S5**). No significant interactions between experiment and treatment were detected (**Supplementary Tables S4**, **S5**).

**Figure 1:**
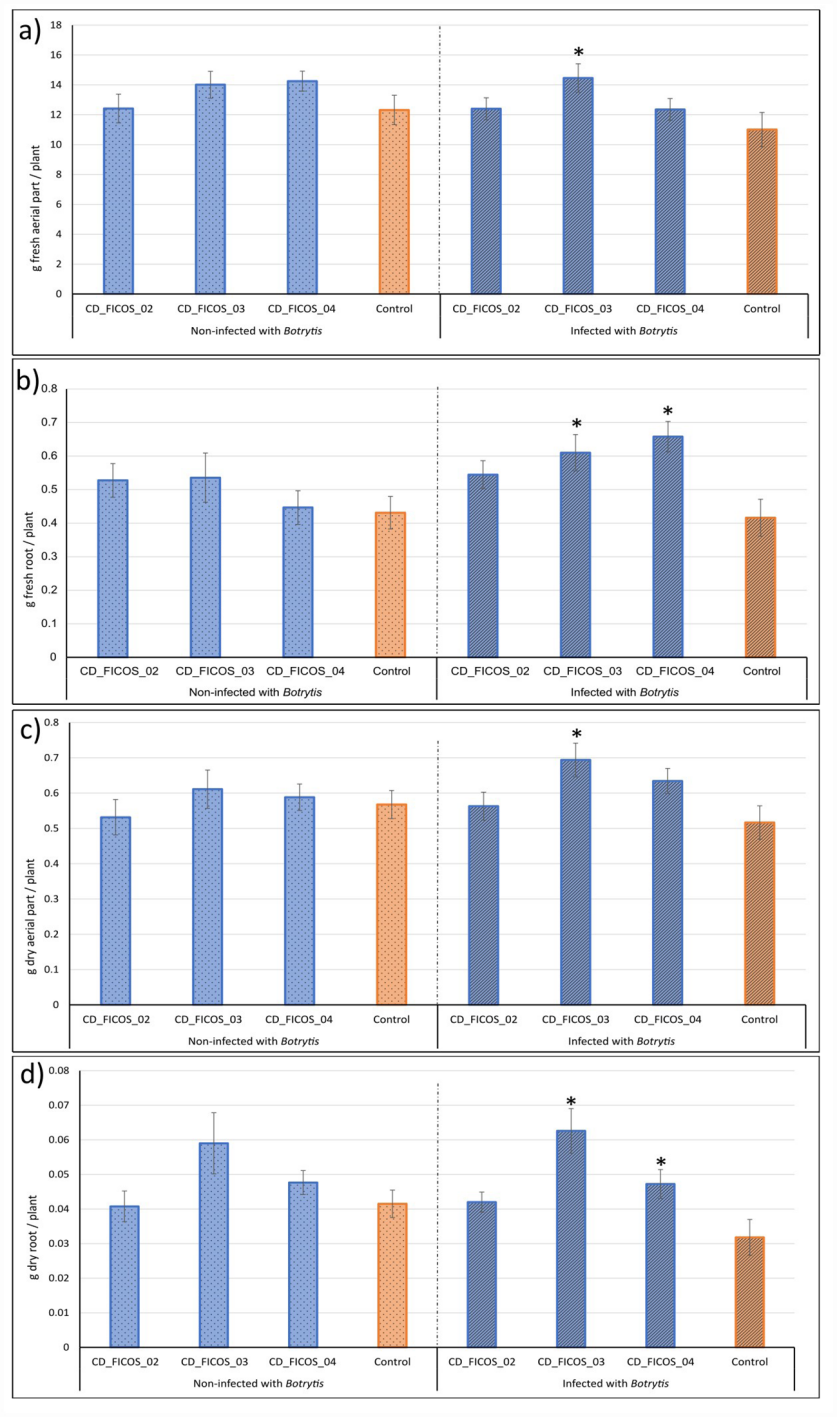
Effect of inoculation on tomato plants under normal conditions and after infection with *Botrytis cinerea*. Mean aerial fresh weight **(a)**, mean root fresh weight **(b)**, mean aerial dry weight **(c)** and mean root dry weight **(d)** of tomato plants inoculated with strains CD_FICOS_02, CD_FICOS_03 and CD_FICOS_04 infected and non-infected with *Botrytis cinerea* compared to the non-inoculated infected control and non-inoculated non-infected control respectively. Values represent the mean of 5 biological replicates per treatment and per experiment of the two experiments, and bars indicate the standard error (SE). Two-way analysis of variance (ANOVA) for the treatment and experiment factor was performed, followed by a Dunnet’s test for the treatment factor. Asterisks indicate significative differences (P <0.05) between the mean values of each treatment and its corresponding control. No significance was found for the experiment factor or for the treatment*experiment interaction.

Treatments infected with *B. cinerea* and inoculated with strain CD_FICOS_03 showed significant improvements in all observed variables compared to the infected and non-inoculated control. Specifically, there were increases of 31% in fresh aerial biomass, 46% in fresh root biomass, 34% in dry aerial biomass and 97% in dry root biomass. In contrast, treatments infected and inoculated with strain CD_FICOS_04 exhibited significant increases in dry and fresh root biomass compared to the infected and non-inoculated control, with increases of 58% and 48% respectively (**Figure 1**; **Supplementary Tables S6**, **S7**).

### 3.3 *Botrytis cinerea* leaf injury

**Figure 2:**
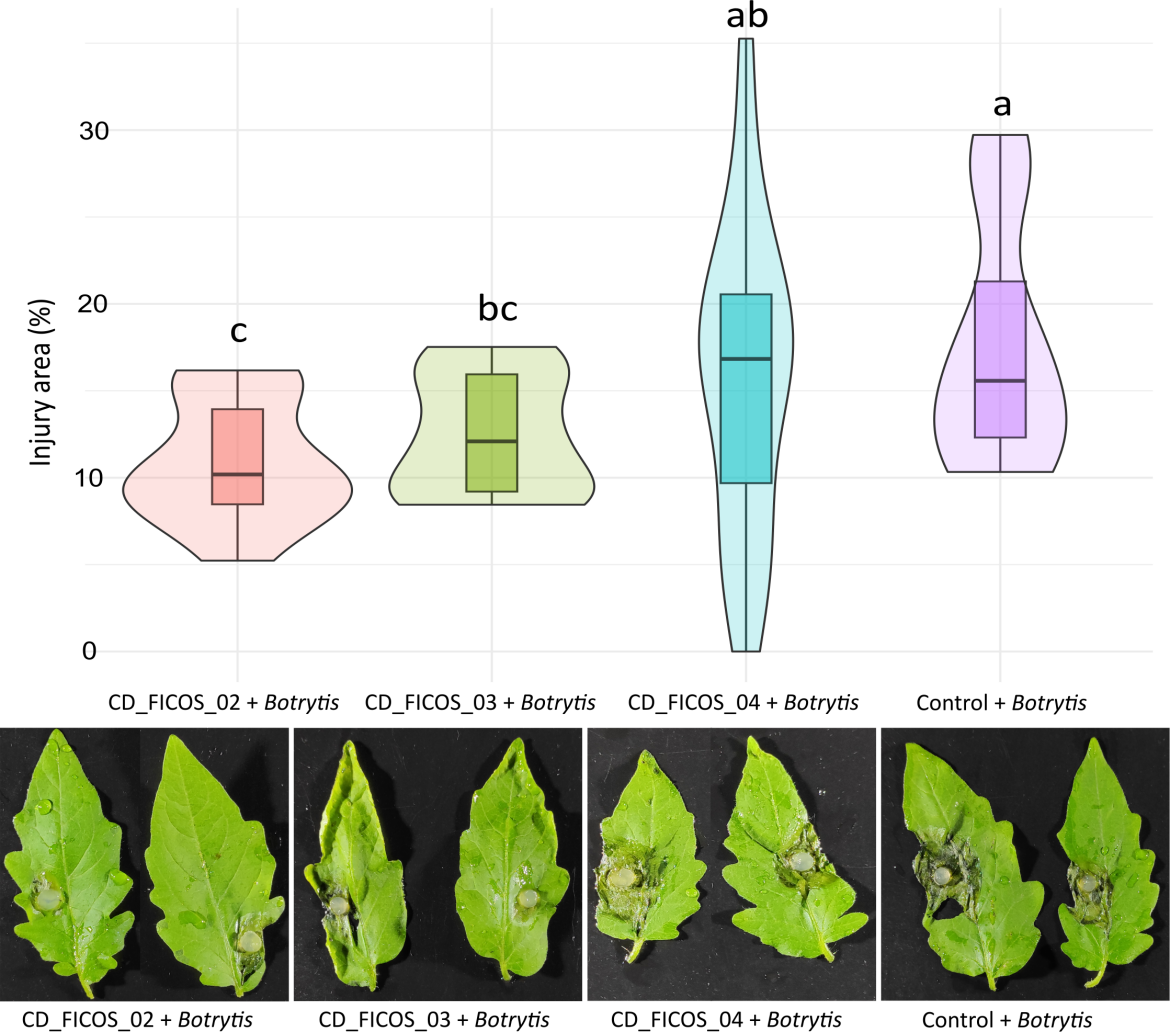
Lesions produced by *Botrytis cinerea* in tomato leaves. Boxplot and kernel density estimation (KDE) to compare the distribution of values between treatments. The boxplot visualizes the median and quartiles, while the KDE provides a smooth estimate of the underlying distribution of the data. Values represent the mean of 10 biological replicates (2 leaves of 5 plants) per treatment and per experiment from two experiments, and the bars indicate the dispersion of the data. The degree of injury is expressed as the percentage of leaf area affected by *Botrytis cinerea* infection. Control: Non-inoculated control; CD_FICOS_02: Inoculated with *Peribacillus frigoritolerans* CD_FICOS_02; CD_FICOS_03: Inoculated with *Pseudomonas canadensis* CD_FICOS_03; CD_FICOS_04: Inoculated with *Azotobacter chroococcum* CD_FICOS_04. The different letters signify significant differences (P <0.05) according to a Kruskal-Wallis test for the factor experiment. The Kruskal-Wallis test for the experiment factor showed no significant differences between experiment 1 and 2.

Signs of injury caused by *B. cinerea* infection were exclusively observed in treatments infected with the fungus. ANOVA revealed that the variable experiment did not produce significant differences on the dependent variable lesion. Whereas the treatment with the inoculation of PGPR strains did produce significant differences (**Supplementary Table S8**). On the other hand, no significant interactions between experiment and treatment were detected (**Supplementary Table S8**). Within the infected treatments, a reduction in lesion area was observed in treatments inoculated with strains CD_FICOS_02 and CD_FICOS_03 compared to the non-inoculated control with a percentage decrease of 37 and 29% respectively. However, inoculation with strain CD_FICOS_04 failed to reduce the lesion (**Figure 2**; **Supplementary Table S9**).

### 3.4 Hydrogen peroxide and malondialdehyde content

**Figure 3:**
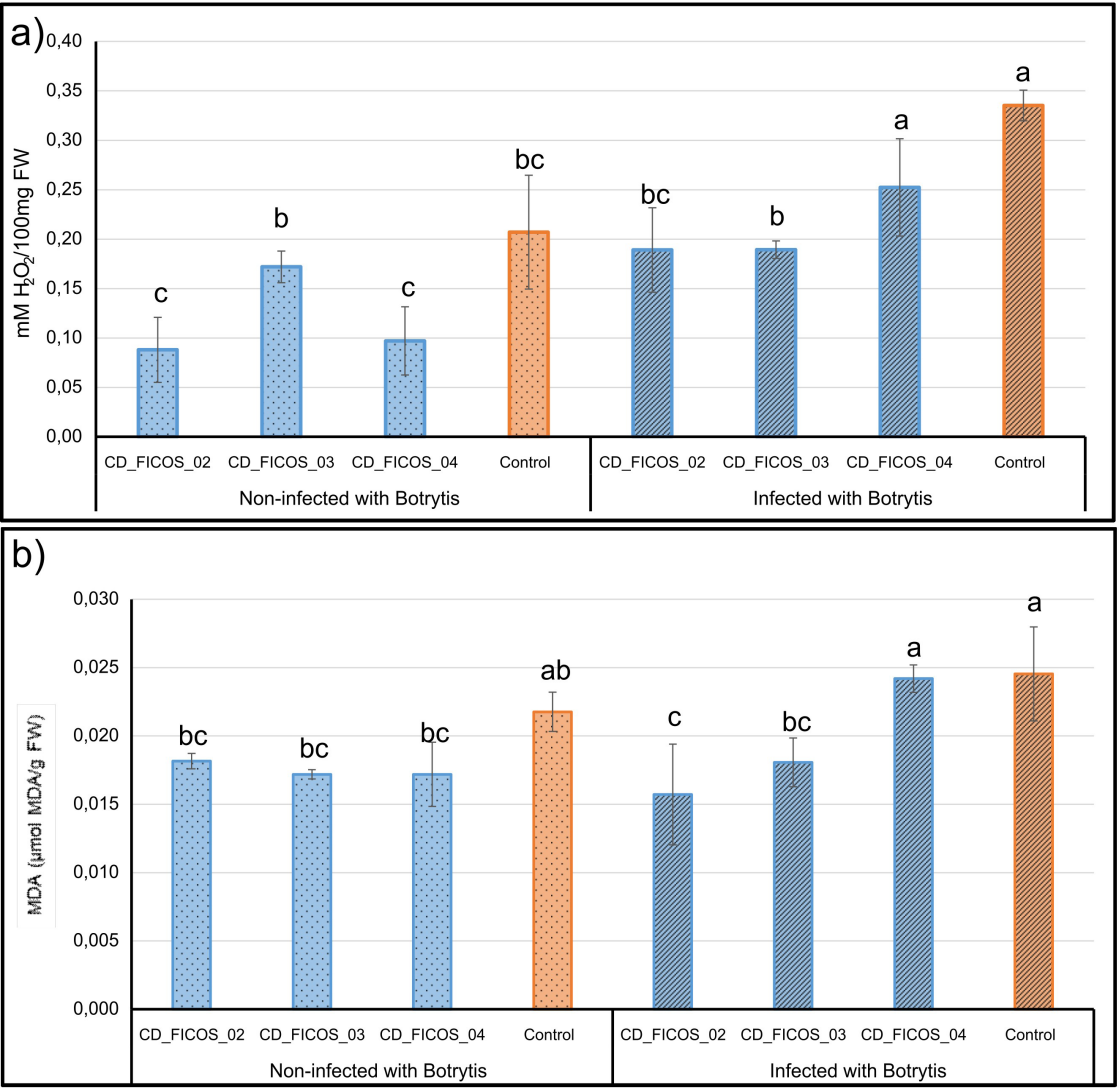
Hydrogen peroxide (H_2_O_2_) and malondialdehyde (MDA) content in leaves of *Botrytis cinerea* infected and non-infected tomato plants. Mean H_2_O_2_ content **(a)** and MDA content **(b)** of tomato plants inoculated with strains CD_FICOS_01, CD_FICOS_02 and CD_FICOS_03 infected and non-infected with *Botrytis cinerea* compared to the non-inoculated infected control and non-inoculated non-infected control respectively. Values represent the mean of 3 biological replicates per treatment and per experiment of the two experiments, and bars indicate the standard error (SE). Two-way analysis of variance (ANOVA) for the treatment and experiment factor was performed, followed by a Tukey test for the treatment factor. The different letters signify significant differences (P <0.05). No significance was found for the experiment factor or for the treatment*experiment interaction.

ANOVA showed that the variable experiment did not produce significant differences in the dependent variables hydrogen peroxide (H_2_O_2_) and malondialdehyde (MDA) content, while the treatment received did have a significant effect (**Supplementary Table S10**). No significant experiment-treatment interactions were detected (**Supplementary Table S10**). Biotic stress induced by infection led to an increase in H_2_O_2_ contents, with a 61.91% increase in infected control plants compared to non-infected control plants (**Figure 3**; **Supplementary Table S11**). Inoculation with strains CD_FICOS_02 and CD_FICOS_03 effectively decreased H_2_O_2_ levels in infected plants, resulting in a 43.62% reduction compared to the infected and non-inoculated controls. In addition, plants infected and inoculated with these PGPR strains showed no significant increase in H_2_O_2_ content compared to non-infected treatments. (**Figure 3**; **Supplementary Tables S10**, **S11**).

Overall, the differences in MDA content between treatments are relatively minor compared to those in H_2_O_2_ content, although a similar pattern is maintained (**Figure 3**; **Supplementary Table S11**). In contrast to the H_2_O_2_ results, no significant increase in MDA content is observed in the non-inoculated and infected control compared to the non-inoculated and non-infected control. However, a reduction is noted in the treatments inoculated with strains CD_FICOS_02 and CD_FICOS_03, showing decreases of 35.93% and 26.39% respectively, compared to the control within the infected treatments (**Figure 3**; **Supplementary Tables S10**, **S11**).

### 3.5 Induced systemic resistance gene expression

To assess the effect of inoculation with PGPR and/or infection with *B. cinerea* on triggering the plant response through induced systemic resistance (ISR), the expression of four genes related to the synthesis or response the hormones salicylic acid (SA), jasmonic acid (JA) and ethylene (ET), involved in plant defense was evaluated.

ANOVA showed that the experiment variable did not produce significant differences on the dependent variable gene expression, while the treatment received did produce significant differences (**Supplementary Table S12**). No significant interactions were detected between experiment and treatment factors (**Supplementary Table S12**).

**Figure 4:**
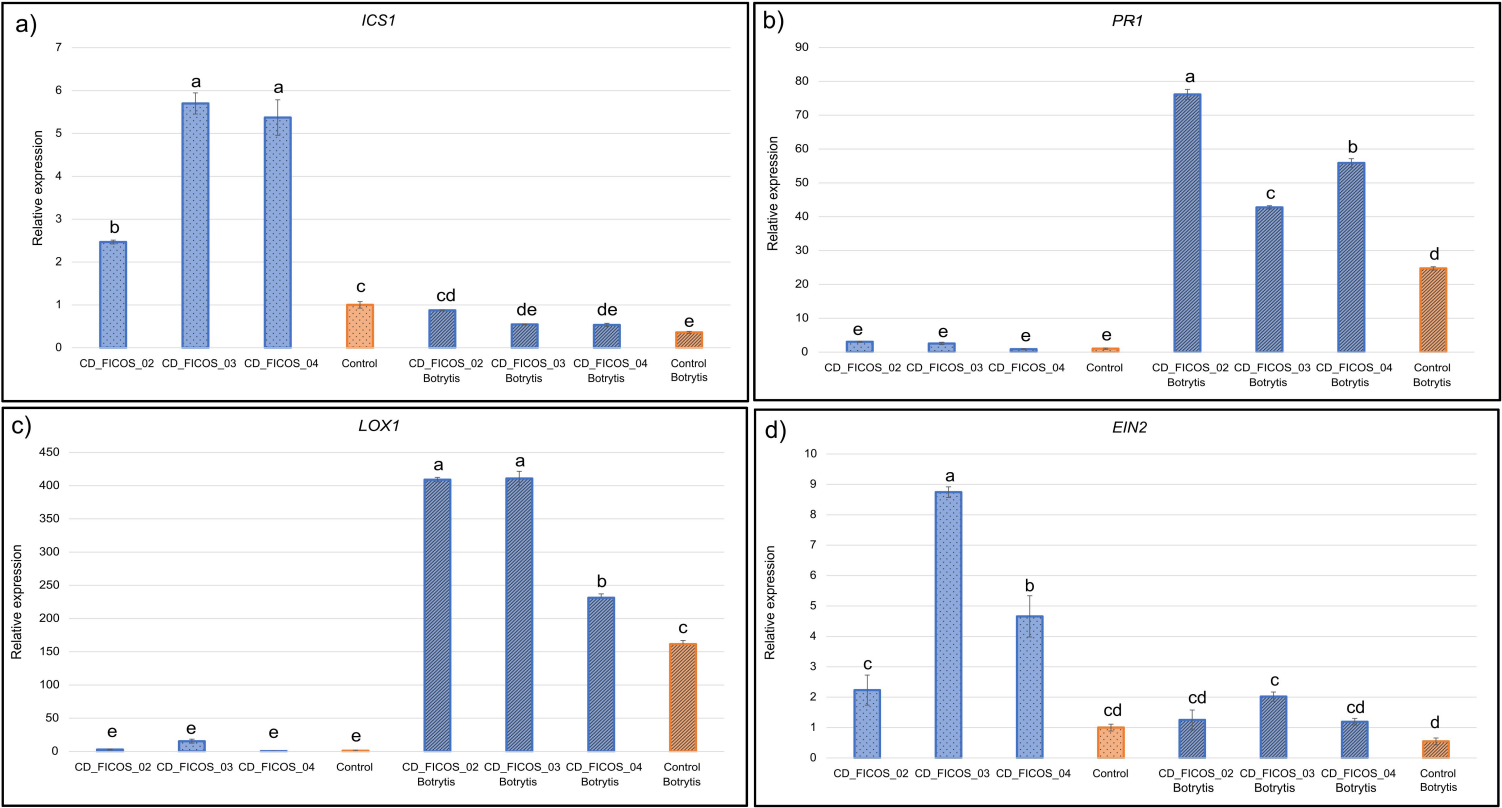
RT-qPCR analysis of defense genes expression in tomato plants. Control: Non-inoculated control; CD_FICOS_02: Inoculated with *Peribacillus frigoritolerans* CD_FICOS_02; CD_FICOS_03: Inoculated with *Pseudomonas canadensis* CD_FICOS_03; CD_FICOS_04: Inoculated with *Azotobacter chroococcum* CD_FICOS_04; *Botrytis:* treatments infected with *Botrytis cinerea.* The genes analysed were *Isochorismate Synthase 1* (*ICS1*) **(a)**, *Pathogenesis-related Protein 1* (*PR-1*) **(b)**, *lipoxygenase 1* (*LOX1*) **(c)** and *Ethylene Signaling Protein* (*EIN2*) **(d)**. The measurements were referenced to the value obtained for the non-inoculated and non-infected plants (Control) (2–ΔΔCt = 1). The tomato Actin gene was used as an internal reference gene. Values represent the mean of 3 technical replicates for each of the 3 biological replicates per treatment and per experiment of two experiments, and the bars indicate the standard error (SE). Two-way analysis of variance (ANOVA) for the treatment and experiment factor was performed, followed by a Tukey test for the treatment factor. The different letters signify significant differences (P <0.05). No significance was found for the experiment factor or for the treatment*experiment interaction.

The expression of the *ICS1* gene, implicated in SA synthesis, was reduced in response to *B. cinerea* infection. In non-infected treatments, inoculation with the different strains individually increased the expression of this gene. While in infected treatments, only inoculation with strain CD_FICOS_02 caused an up-regulation of *ICS1* expression compared to the non-inoculated infected control (**Figure 4**; **Supplementary Table S12**).

The *PR-1* gene, involved in the SA pathway, was overexpressed in response to fungal infection (**Figure 4**; **Supplementary Table S12**). Additionally, among the infected treatments, all strains promoted *PR-1* overexpression, with CD_FICOS_02 showing the strongest effect.

The *LOX1* gene, associated with JA synthesis, followed a similar pattern to *PR-1*. It was also overexpressed both in response to *B. cinerea* infection and, in some cases, upon inoculation with PGPR strains. In infected treatments, all strains induced *LOX1* overexpression compared to the control, with CD_FICOS_02 and CD_FICOS_03 having the most pronounced effect (**Figure 4**; **Supplementary Table S12**).

Regarding the *EIN2* gene, involved in the ET pathway, *B. cinerea* infection alone had no effect on its expression. However, PGPR inoculation led to increased *EIN2* expression under certain conditions. Specifically, in the absence of infection, CD_FICOS_04 and especially CD_FICOS_03 up-regulated *EIN2*. In infected plants, only CD_FICOS_03 induced gene overexpression (**Figure 4**; **Supplementary Table S12**).

In summary, *B. cinerea* infection increased *ICS1* gene expression while reducing *PR-1* and *LOX1* expression. Meanwhile, inoculation with PGPRs, in general, increases the expression of the four genes analyzed in almost all cases. Showing a greater relationship between strain CD_FICOS_02 with the overexpression of genes *ICS1* and *PR1* related to the SA; and CD_FICOS_03 with the up-regulation of *LOX1* and *EIN2* related to the JA/ET pathway (**Figure 4**; **Supplementary Table S12**).

## 4 Discussion

The benefits of inoculation with plant growth-promoting rhizobacteria (PGPR) leading to increased plant production are well-known and documented (Barquero et al., 2022; Mazuecos-Aguilera et al., 2024). In addition, certain bacterial strains have been identified as contributing to plant defense against pathogens, either directly or by triggering plant defense mechanisms (Poveda et al., 2020, 2022). In this study, we evaluated three PGPR strains to activate Induced Systemic Resistance (ISR) in tomato and enhance defense against *Botrytis cinerea* infection.

The three PGPR strains tested in this study exhibited various plant growth-promoting (PGP) traits, including phytohormones production and the improvement of essential nutrients absorption, resulting in an improved nutritional status and consequently an increase in biomass production. This was evident in the hydroponic assay where axenic substrate was used. However, in the tomato assay, with non-sterile substrate, significant biomass increases were only observed in plants infected with *B. cinerea* and inoculated with strains CD_FICOS_03 and CD_FICOS_04. This observation aligns with previous studies (Choudhary et al., 2016; Majeed et al., 2018) confirming that the benefits of PGPRs are enhanced under different stress conditions, as would correspond to the case of an axenic substrate or infection by a phytopathogen. The strain *P. canadensis* CD_FICOS_03 shows the highest biomass increase in the tomato assay, being the most efficient phosphorus solubilizer in addition to exhibiting good values for the other PGP characteristics. The PGP effect of different *Pseudomonas* species has been described in numerous studies (Dorjey et al., 2017). *A. chroococcum* CD_FICOS_04 shows an increase in root biomass in tomato plants, consistent with reports of the genus *Azotobacter* enhancing nitrogen fixation and conferring additional crop benefits (Wani et al., 2016).

Interestingly, while CD_FICOS_04 was unable to reduce the lesions caused by the phytopathogenic fungus, it induces an increase in biomass in infected treatments compared to the non-inoculated infected control. Conversely, the strain CD_FICOS_02, which does not increase biomass production, is the one that most reduces the lesion caused by *B. cinerea* infection. This suggests that the reduction of injury is not related to the nutritional enhancement by the PGPR capabilities of the strains used but is achieved by another pathway. One possible way PGPR strains may mitigate *B. cinerea* infection is through the production of indole-3-acetic acid (IAA) (Nafisi et al., 2015). However, despite similar *in vitro* IAA production levels among the three strains, only CD_FICOS_02 and CD_FICOS_03 reduced *B. cinerea* lesions, while CD_FICOS_04 failed to mitigate the lesion. Thus, IAA production does not appear to be the primary mechanism behind lesion reduction.

Therefore, we evaluated whether the infection reduction achieved with the inoculation of CD_FICOS_02 and CD_FICOS_03 could be attributed to ISR activation. In general, inoculation with PGPRs, especially CD_FICOS_02 and CD_FICOS_03, resulted in upregulation of ISR-related genes, including those involved in the salicylic acid (SA) pathway (*ICS1* and *PR1*), and the jasmonic acid/ethylene (JA/ET) pathway (*LOX1* and *EIN2*) (Poveda et al., 2020). Typically, ISR is considered independent of SA and devoid of pathogenesis-related (PR) protein accumulation. However, a positive SA-JA interaction and accumulation of PR proteins in ISR, as observed in the present work, has been found in other works (Wang et al., 2016; Tsalgatidou et al., 2023), suggesting that simultaneous induction of both SA and JA signaling occurs. The activation of ISR through both hormonal pathways is attributed to the diverse elicitors produced by PGPR, capable of triggering distinct defensive response pathways (Wu et al., 2018; Tsalgatidou et al., 2023).

Inoculation with the strain CD_FICOS_02, which reduced lesion formation by 37%, induced a more pronounced overexpression than the other strains of the *PR-1* gene in the presence of *B. cinerea* and *ICS1* in both absence and presence of *B. cinerea*. This suggests a heightened induction of ISR via the SA-dependent pathway. The upregulation of *ICS1* allows for increased synthesis of isochorismate, a precursor of SA, from chorismate. Consequently, elevated SA levels induce the expression of response genes, including *PR1*, which can inhibit the pathogen growth and dissemination by sequestering pathogen sterols (Qi et al., 2018; Poveda et al., 2020). Specifically, antimicrobial activity of a homologue of *PR-1* against *B. cinerea* has been observed in tobacco plants (Kiba et al., 2007). Biocontrol of *B. cinerea* through the activation of the SA-dependent pathway by inoculation with *Bacillus*, phylogenetically close to the genus *Peribacillus* to which CD_FICOS_02 belongs, has been previously reported in several assays (Poveda et al., 2020; Roca-Couso et al., 2021). Similarly, to our work, Yoshida et al. (2019) demonstrated that inoculation with a *B. thuringiensis* strain was effective against *B. cinerea* in tomato by increasing the expression of the *PR1* gene. Inoculation with CD_FICOS_02 not only induces ISR but also triggers a priming phenomenon, particularly noticeable in the upregulation of genes *ICS1* and *LOX1*, which intensifies upon exposure to *B. cinerea*. Consequently, prior to *B. cinerea* infection, inoculation would cause an accumulation of transcripts of these genes below the threshold for effective resistance but allowing for accelerated defense activation upon *B. cinerea* infection (Akram et al., 2008; Tsalgatidou et al., 2023).

On the other hand, treatments inoculated with the CD_FICOS_03 strain, which reduced lesion formation by 29%, induced stronger expression of *LOX1* and *EIN2*, involved in the JA/ET signaling pathway (Poveda et al., 2020). *LOX1* upregulation leads to increased lipid hydroperoxides (HPOTs), precursors of JA, compounds with antimicrobial activity, and triggers the hypersensitivity response (HR) (Porta and Rocha-Sosa, 2002). Meanwhile, *EIN2* is a key component of ET signaling, which has been shown to a critical role in *B. cinerea* resistance, as *Arabidopsis* mutants lacking *EIN2* exhibit increased susceptibility to the pathogen (Thomma et al., 1999). JA and ET pathways are interconnected, whit ET inducing JA biosynthesis, and vice versa. Additionally, there also seems to be a transcriptional interaction of the ET, JA, and SA signaling pathways, where ET acts as a modulator of the other two (AbuQamar et al., 2017). Thus, ISR induction by the *P. canadensis* CD_FICOS_03 is mainly carried out via the JA/ET pathway, which aligns with findings in other strains of the genus that manage to reduce damage caused by *B. cinerea* through this same signaling pathway (Poveda et al., 2020). Akram et al. (2008) and Ongena et al. (2004) also observed that the BTP1 strain of *P. putida* reduces infection caused by *B. cinerea* in tomato and bean by inducing lipoxygenase (LOX) activity, the same that observed in our work.

In contrast, *A. chroococcum* CD_FICOS_04 induced the lowest gene overexpression, consistent with its inability to control *B. cinerea* infection. Some *Azotobacter* strains have been reported to suppress fungal pathogens through siderophore production and the consequent iron sequestration, necessary for the growth and germination of *B. cinerea* spores, or through the production of different antibiotics, rather than ISR activation (Hindersah et al., 2021; Muslim et al., 2021; Roca-Couso et al., 2021). In this study, there is no direct confrontation between the CD_FICOS_03 strain of *Azotobacter* and *B. cinerea* as they were applied to the root and leaf respectively and therefore direct biocontrol of the disease was not part of the experimental design.

Finally, our findings confirm that *B. cinerea* infection leads to an increase in hydrogen peroxide (H_2_O_2_) levels, indicating an elevation in reactive oxygen species (ROS) production, consistent with previous studies (Kazerooni et al., 2021). While ROS play a crucial role in stress signaling, their excessive accumulation can result in oxidative damage to plant tissues. In contrast, inoculation with strains CD_FICOS_02 and CD_FICOS_03 effectively reduced H_2_O_2_ and malondialdehyde (MDA) levels in *B. cinerea*-infected plants, thereby preventing excessive ROS accumulation and mitigating oxidative stress. This reduction in ROS levels correlates with the observed decrease in lesion size, reinforcing the protective role of these PGPR strains. The ability of PGPR to reduce ROS accumulation under various stress conditions has been widely reported (Kazerooni et al., 2021; Geng et al., 2022). In this context, the observed ACC deaminase activity in CD_FICOS_02 and CD_FICOS_03 appears to be closely linked to their ability to mitigate infection severity and oxidative stress, as measured by reductions in H_2_O_2_ and MDA levels. ACC deaminase activity likely contributes to this effect by lowering ethylene levels under biotic stress conditions, thereby preventing excessive ROS production (Naing et al., 2021; Sofy et al., 2021; Chandwani and Amaresan, 2022).

## 5 Conclusions

Soil inoculation with the PGPR strains *Peribacillus frigoritolerans* CD_FICOS_02 and *Pseudomonas canadensis* CD_FICOS_03 demonstrated capabilities to reduce damage caused by *Botrytis cinerea* infection and oxidative stress. Both strains caused overexpression of Induced Systemic Resistance (ISR) related genes, involved in the salicylic acid (SA), jasmonic acid (JA) and ethylene (ET) hormonal pathways. Strain CD_FICOS_02 was more prominent in the overexpression of SA-related genes and CD_FICOS_03 in JA/ET-related genes. Additionally, in *B. cinerea* infected plants, inoculation with *P canadensis* CD_FICOS_03 and *Azotobacter chroococcum* CD_FICOS_04 strains, although the latter is not able to reduce lesions, resulted in a significant increase in tomato plant biomass production. These results suggest that the different PGPR strains evaluated can effectively enhance plant defense mechanisms against *B. cinerea* attack, but without a clear correlation between PGPR conditions and the mechanism of plant protection against disease.

## Data Availability

The datasets presented in this study can be found in online repositories. The names of the repository/repositories and accession number(s) can be found in the article/**Supplementary Material**.
