# Supplementary material for: Plant growth-promoting rhizobacteria enhanced induced systemic resistance of tomato against Botrytis cinerea phytopathogen
Source: Front Plant Sci. 2025 Apr 15;16:1570986. doi: 10.3389/fpls.2025.1570986 (PMC12038444; doi:10.3389/fpls.2025.1570986)
Supplement: Supplementary file 1 [file DataSheet1.docx]

Supplementary Material

**Supplementary Table 1**. Primers used in RT-qPCR.

| Primer | Sequence (5′-3′) | Gene and gene function |
| --- | --- | --- |
| Act-Sl-F | CACCACTGCTGAACGGGAA | Endogenous tomato gene |
| Act-Sl-R | GGAGCTGCTCCTGGCAGTTT |  |
| ICS1-Sl-F | GTTCCTCTCCAAGAATGTCC | Synthesis gene of SA in tomato  (isochorismate synthase1) |
| ICS1-Sl-R | TCCTTCAAGCTCATCAAACT |  |
| PR-1-Sl-F | CCTCAAGATTATCTTAACGCTC | Response gene to SA in tomato  (pathogenesis-related 1) |
| PR-1-Sl-R | TACCATTGCTTCTCATCAACC |  |
| LOX1-Sl-F | GCCTCTCTTCTTGATGGAG | Synthesis gene of JA in tomato  (lipoxygenase 1) |
| LOX1-Sl-R | GTAGTGAGCCACTTCTCCAA |  |
| EIN2-Sl-F | GTTGCTAAGTGATGCTGTA | Response gene to JA/ET in tomato  (ethylene-insensitive 2) |
| EIN2-Sl-R | CGCTCAAGCATGCTGGGCC |  |


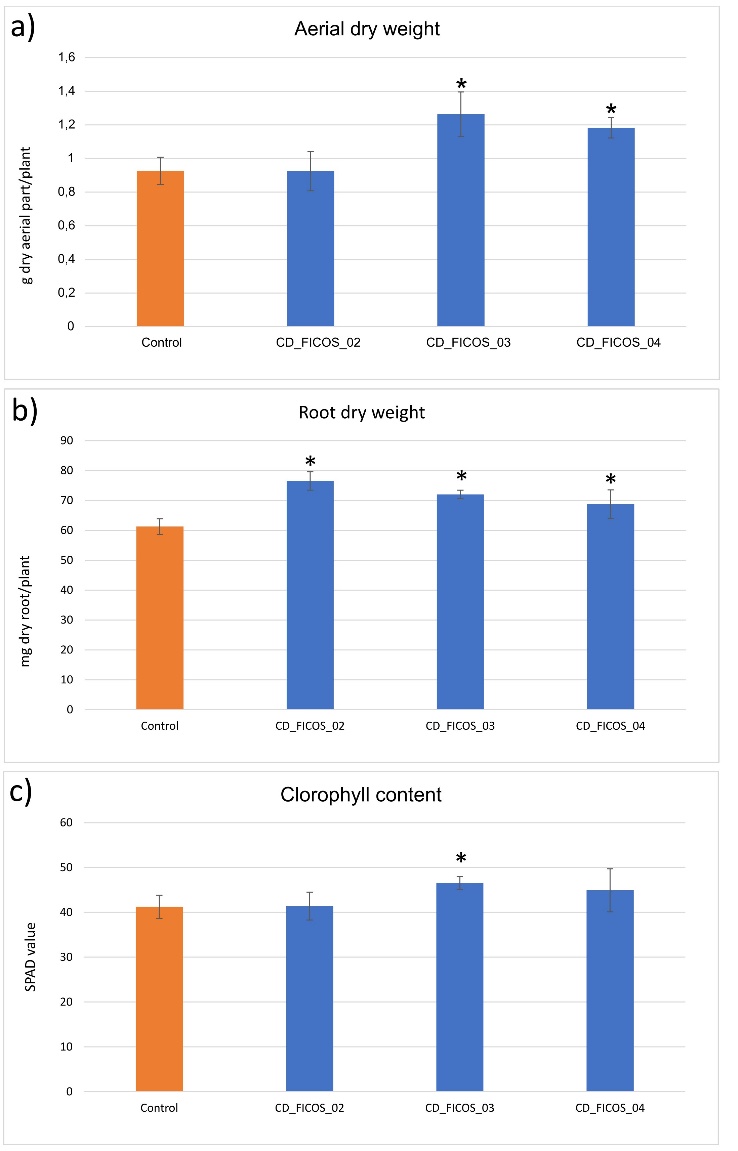


**Supplementary Figure 1.** Effect of inoculation on barley plants in the in-plant test in hydroponic conditions. Mean aerial dry weight (A), mean root dry weight (B) and mean clorofile content (C) of barley plants inoculated with strains CD_FICOS_02, CD_FICOS_03 and CD_FICOS_04 compared to the non-inoculated control. Values represent the mean of 2 experimental replicates with 5 biological replicates per treatment for each experiment, and the bars indicate the standard error (SE). A two-factor analysis of variance (ANOVA) was performed, the factors were experiment and treatment. A Dunnet's test was included as a post-hoc analysis. Asterisks indicate significative differences (P <0.05) between the mean values of each treatment and the control. No significance was found for the experiment factor or for the treatment*experiment interaction.

**Supplementary Table 2**. Two-way ANOVA for aerial and root dry biomass and chlorophyll content of barley plants in hydroponic trial for treatment and experimental factors.

| Source variation | D.F. | Aerial dry | | Root dry | | Clorophyll content | |
| --- | --- | --- | --- | --- | --- | --- | --- |
|  |  | RMS | F | RMS | F | RMS | F |
| Experiment | 1 | 0.018 | 1.695ns | 9.000 | 0.800ns | 9.138 | 0.880ns |
| Treatment | 3 | 0.245 | 22.992* | 165.083 | 14.674* | 55.588 | 5.354* |
| Treatm*Experim | 3 | 0.005 | 0.436ns | 8.500 | 0.756ns | 6.984 | 0.673ns |
| Error | 33 | 0.011 |  | 11.250 |  | 10.382 |  |

Asterisks indicate significance<0.05; ns, not significant. D.F., degrees of freedom; RMS root mean square.

**Supplementary Table 3**. Dunnet’s test results for the variable aerial and root dry biomass and chlorophyll content of barley plants in hydroponic trial and for the factor treatment.

| Variable | (I) Treatment | (J) Treatment | Mean difference (I-J) | Desv. Error | Sig. | 95% confidence interval | |
| --- | --- | --- | --- | --- | --- | --- | --- |
|  |  |  |  |  |  | Lower limit | Upper limit |
| Aerial dry biomass | CD_FICOS_02 | Control | -0.0012 | 0.05159 | 1.000 | -0.1306 | 0.1281 |
|  | CD_FICOS_03 | Control | 0.3375* | 0.05159 | <0.001 | 0.2082 | 0.4668 |
|  | CD_FICOS_04 | Control | 0.2562* | 0.05159 | <0.001 | 0.1269 | 0.3856 |
| Root dry biomass | CD_FICOS_02 | Control | 15.2500* | 2.37171 | <0.001 | 8.4203 | 22.0797 |
|  | CD_FICOS_03 | Control | 10.7500* | 2.37171 | 0.005 | 3.9203 | 17.5797 |
|  | CD_FICOS_04 | Control | 7.5000* | 2.37171 | 0.033 | .6703 | 14.3297 |
| Clorophylle content | CD_FICOS_02 | Control | 0.2000 | 1.61104 | 0.999 | -3.8383 | 4.2383 |
|  | CD_FICOS_03 | Control | 5.3250* | 1.61104 | 0.008 | 1.2867 | 9.3633 |
|  | CD_FICOS_04 | Control | 3.7125 | 1.61104 | 0.076 | -0.3258 | 7.7508 |

Asterisks indicate significance<0.05

**Supplementary Table 4**. Two-way ANOVA for biomass produced by non-infected tomato plants for treatment and experiment factors.

| Source variation | D.F. | Treatments non-infected with *Botrytis* | | | | | | | |
| --- | --- | --- | --- | --- | --- | --- | --- | --- | --- |
|  |  | Fresh | | | | Dry | | | |
|  |  | Aerial | | Root | | Aerial | | Root | |
|  |  | RMS | F | RMS | F | RMS | F | RMS | F |
| Experiment | 1 | 1.767 | 0.205ns | 1.460E-5 | 0.001ns | 0.008 | 0.320ns | 9.347E-5 | 0.343ns |
| Treatment | 3 | 9.658 | 1.121ns | 0.007 | 0.326ns | 0.021 | 0.795ns | 0.001 | 2.541ns |
| Treatm*Experim | 3 | 1.875 | 0.218ns | 0.011 | 0.476ns | 0.058 | 2.229ns | 0.000 | 1.602ns |
| Error | 33 | 8.615 |  | 0.022 |  | 0.026 |  | 0.000 |  |

Asterisks indicate significance<0.05; ns, not significant. D.F., degrees of freedom; RMS root mean square.

**Supplementary Table 5**. Two-way ANOVA for biomass produced by infected tomato plants for treatment and experiment factors.

| Source variation | D.F. | Treatments infected with *Botrytis* | | | | | | | |
| --- | --- | --- | --- | --- | --- | --- | --- | --- | --- |
|  |  | Fresh | | | | Dry | | | |
|  |  | Aerial | | Root | | Aerial | | Root | |
|  |  | RMS | F | RMS | F | RMS | F | RMS | F |
| Experiment | 1 | 1.133 | 0.161ns | 0.007 | 0.420ns | 0.017 | 0.775ns | 3.34E-5 | 0.151ns |
| Treatment | 3 | 21.058 | 2.984* | 0.053 | 2.977* | 0.107 | 4.994* | 0.002 | 7.532* |
| Treatm*Experim | 3 | 1.256 | 0.178ns | 0.009 | 0.507ns | 0.023 | 1.055ns | 4.493E-5 | 0.203ns |
| Error | 33 | 7.056 |  | 0.018 |  | 0.021 |  | 0.000 |  |

Asterisks indicate significance<0.05; ns, not significant. D.F., degrees of freedom; RMS root mean square.

**Supplementary Table 6**. Mean values and percentage increase with respect to the corresponding control of the biomass ±SE produced by tomato plants. The treatment with non-infected and non-inoculated plants (Control) is the control for the treatments with inoculated and non-infected plants. The treatment with infected and non-inoculated plants (Control+*Botrytis*) is the control for the treatments with inoculated and infected plants.

| Treatment | Fresh biomass | | | | Dry biomass | | | |
| --- | --- | --- | --- | --- | --- | --- | --- | --- |
|  | Aerial part | | Root | | Aerial part | | Root | |
|  | g/plant | Increase (%) | g/plant | Increase (%) | g/plant | Increase (%) | mg/plant | Increase (%) |
| CD_FICOS_02 | 12.42±0.96 | 0.80 | 0.53±0.05 | +22.39 | 0.53±0.05 | -6.35 | 40.77±4.42 | -1.74 |
| CD_FICOS_03 | 14.01±0.90 | 13.72 | 0.54±0.07 | +24.26 | 0.61±0.05 | 7.67 | 59±8.81 | +42.17 |
| CD_FICOS_04 | 14.25±0.67 | 15.68 | 0.45±0.05 | +3.63 | 0.59±0.04 | 3.66 | 47.66±3.46 | +14.86 |
| Control | 12.32±0.98 |  | 0.43±0.05 |  | 0.57±0.04 |  | 41.5±3.95 |  |
| CD_FICOS_02 + *Botrytis* | 12.40±0.73 | 12.68 | 0.54±0.04 | +30.80 | 0.56±0.04 | 8.96 | 42±2.89 | +32.08 |
| CD_FICOS_03 + *Botrytis* | 14.46±0.95* | 31.33 | 0.61±0.05* | +46.55 | 0.69±0.05* | 34.29 | 62.6±6.45* | +96.86 |
| CD_FICOS_04 + *Botrytis* | 12.35±0.73 | 12.23 | 0.66±0.05* | +58.02 | 0.63±0.04 | 22.77 | 47.22±4.18* | +48.50 |
| Control + *Botrytis* | 11.01±1.14 |  | 0.42±0.06 |  | 0.52±0.05 |  | 31.8±5.16 |  |

Asterisks indicate significative differences between the mean values of each treatment and its corresponding control, according to Dunnet’s test.

**Supplementary Table 7**. Dunnet’s test results for the variable biomass produced by tomato plants and for the factor treatment.

| Variable | | | (I) Treatment | (J) Treatment | Mean difference (I-J) | Desv. Error | Sig. | 95% confidence interval | |
| --- | --- | --- | --- | --- | --- | --- | --- | --- | --- |
|  |  |  |  |  |  |  |  | Lower limit | Upper limit |
| Treatments infected with *Botrytis* | Fresh | Aerial | CD_FICOS_02+*Botrytis* | Control+*Botrytis* | 1.3956 | 1.19397 | 0.521 | -1.5500 | 4.3411 |
|  |  |  | CD_FICOS_03+*Botrytis* | Control+*Botrytis* | 3.4493* | 1.16067 | 0.015 | 0.5859 | 6.3127 |
|  |  |  | CD_FICOS_04+*Botrytis* | Control+*Botrytis* | 1.3467 | 1.13270 | 0.508 | -1.4476 | 4.1411 |
|  |  | Root | CD_FICOS_02+*Botrytis* | Control+*Botrytis* | 0,0463 | 0,06135 | 0,799 | -0,1065 | 0,1991 |
|  |  |  | CD_FICOS_03+*Botrytis* | Control+*Botrytis* | 0,1772* | 0,06334 | 0,025 | ,00194 | 0,3349 |
|  |  |  | CD_FICOS_04+*Botrytis* | Control+*Botrytis* | 0,1176 | 0,06135 | 0,161 | -0,0352 | 0,2704 |
|  | Dry | Aerial | CD_FICOS_02+*Botrytis* | Control+*Botrytis* | 0.1282 | 0.07099 | 0.191 | -0.0480 | 0.3043 |
|  |  |  | CD_FICOS_03+*Botrytis* | Control+*Botrytis* | 0.1938* | 0.07099 | 0.029 | 0.0176 | 0.3699 |
|  |  |  | CD_FICOS_04+*Botrytis* | Control+*Botrytis* | 0.2415* | 0.07099 | 0.006 | 0.0654 | 0.4177 |
|  |  | Root | CD_FICOS_02+*Botrytis* | Control+*Botrytis* | 0.0102 | 0.00683 | 0.329 | -0.0067 | 0.0271 |
|  |  |  | CD_FICOS_03+*Botrytis* | Control+*Botrytis* | 0.0308* | 0.00665 | <0.001 | 0.0143 | 0.0472 |
|  |  |  | CD_FICOS_04+*Botrytis* | Control+*Botrytis* | 0.0172* | 0.00683 | 0.046 | 0.00027 | 0.0341 |

Asterisks indicate significance<0.05

**Supplementary Table 8.** Kruskall-Wallis tests results for the variable Botrytis lesion on tomato leaf plants and for the factors treatment and experiment separately.

| Summary of Kruskall-Wallis test for independent samples for factor treatment | | | | |  |
| --- | --- | --- | --- | --- | --- |
| Total N | Test statistic | Degrees of freedom | Asymptotic sig. |  |  |
| 71 | 12.648 | 3 | 0.005 |  |  |
| Comparisons by treatment pair | | | | | |
| Sample 1-Sample 2 | Test statistic | Error Desv. | Test statistic Desv. | Sig. | |
| CD_FICOS_02+*Botrytis* – CD_FICOS_03+*Botrytis* | -6.825 | 7.418 | -0.920 | 0.358 | |
| CD_FICOS_02+*Botrytis* – CD_FICOS_04+*Botrytis* | -17.375 | 6.923 | -2.510 | 0.012 | |
| CD_FICOS_02+*Botrytis* – Control+*Botrytis* | 22.325 | 6.923 | 3.225 | 0.001 | |
| CD_FICOS_03+*Botrytis* – CD_FICOS_04+*Botrytis* | -10.550 | 7.050 | -1.496 | 0.135 | |
| CD_FICOS_03+*Botrytis* – Control+*Botrytis* | 15.500 | 7.050 | 2.199 | 0.028 | |
| CD_FICOS_04+*Botrytis* – Control+*Botrytis* | 4.950 | 6.527 | 0.758 | 0.448 | |

| Summary of Kruskall-Wallis test for independent samples for factor experiment | | | | |  |
| --- | --- | --- | --- | --- | --- |
| Total N | Test statistic | Degrees of freedom | Asymptotic sig. |  |  |
| 71 | 1.611 | 2 | 0.447 |  |  |
| Comparisons by treatment pair | | | | | |
| Sample 1-Sample 2 | Test statistic | Error Desv. | Test statistic Desv. | Sig. | |
| -first | -26.286 | 20.933 | -1.256 | 0.209 | |
| -second | -26.457 | 20.933 | -1.264 | 0.206 | |
| first-second | -0.171 | 4.934 | -0.035 | 0.972 | |

**Supplementary Table 9**. Injury area caused by *B. cinerea* ±SE on tomato leaves. Expressed as the percentage of the affected area in relation to the total leaf area.

| Treatment | Injury área (%) | Injury reduction rate (%) |
| --- | --- | --- |
| CD_FICOS_02 + *Botrytis* | 10.95±0.87* | -37.81 |
| CD_FICOS_03 + *Botrytis* | 12.42±0.9* | -29.60 |
| CD_FICOS_04 + *Botrytis* | 15.89±1.98 | -0.96 |
| Control + *Botrytis* | 17.61±1.53 |  |

Asterisks indicate signifactive differences between the mean values of each treatment and its corresponding control, according to Kruskall-Wallis test

**Supplementary Table 10**. Two-way ANOVA for H_2_O_2_ and MDA content for treatment and experiment factors.

| Source variation | D.F. | H_2_O_2_ | | MDA | |
| --- | --- | --- | --- | --- | --- |
|  |  | RMS | F | RMS | F |
| Experiment | 1 | 0.002 | 1.152ns | 6.089E-7 | 0.088ns |
| Treatment | 7 | 0.031 | 21.340* | 6.284E-5 | 9.067* |
| Treatm*Experim | 7 | 0.000 | 0.152ns | 4.215E-7 | 0.061ns |
| Error | 30 | 0.001 |  | 6.931E-6 |  |

Asterisks indicate significance<0.05; D.F., degrees of freedom; RMS root mean square.

**Supplementary Table 11**. H_2_O_2_ and MDA content ±SD in tomato leaves.

| Treatment | H_2_O_2_ content | | MDA content | |
| --- | --- | --- | --- | --- |
|  | mM/100mg FW | Increase (%) (with regard to) | µmol/g FW | Increase (%) (with regard to) |
| CD_FICOS_02 | 0.088±0.026 c | -57.48 (control) | 1.82E-02±4.84E-04 bc | -16.55 (control) |
| CD_FICOS_03 | 0.172±0.059 b | -16.96 (control) | 1.72E-02±2.90E-04 bc | -21.00 (control) |
| CD_FICOS_04 | 0.097±0.005 c | -53.12 (control) | 1.72E-02±2.03E-03 bc | -21.00 (control) |
| Control | 0.207±0.075 bc |  | 2.18E-02± 1.6E-03 ab |  |
| CD_FICOS_02 + *Botrytis* | 0.189±0.024 bc | -43.62 (control+*Botrytis*) | 1.57E-02±4.12E-03 c | -35.93 (control+*Botrytis*) |
| CD_FICOS_03 + *Botrytis* | 0.189±0.012 b | -43.62 (control+*Botrytis*) | 1.81E-02±5.48E-04 bc | -26.39 (control+*Botrytis*) |
| CD_FICOS_04 + *Botrytis* | 0.252±0.076 a | -24.73 (control+*Botrytis*) | 2.42E-02±1.12E-03 a | -1.40 (control+*Botrytis*) |
| Control + *Botrytis* | 0.335±0.005 a | +61.91 (control) | 2.45E-02±3.84E-03 a | +12.75 (control) |

The different letters signify significant differences, according to a Tukey test.

**Supplementary Table 12**. Two-way ANOVA for the expression levels of the genes analysed in tomato plants for treatment and experiment factors.

| Source variation | D.F. | *ICS1* | | *PR1* | | *LOX1* | | *EIN2* | |
| --- | --- | --- | --- | --- | --- | --- | --- | --- | --- |
|  |  | RMS | F | RMS | F | RMS | F | RMS | F |
| Experiment | 1 | 0.009 | 0.434ns | 0.150 | 0.197 ns | 29.208 | 0.755 ns | 0.032 | 0.205ns |
| Treatment | 7 | 13.218 | 650.891* | 2514.241 | 3309.341* | 141531.803 | 3657.501* | 20.341 | 129.238* |
| Treat*Exp | 7 | 0.044 | 2.188ns | 0.384 | 0.506 ns | 9.383 | 0.242 ns | 0.073 | 0.466 ns |
| Error | 8 | 0.020 |  | 0.760 |  | 38.696 |  | 0.157 |  |

Asterisks indicate significance<0.05; D.F., degrees of freedom; RMS root mean square.
